# DhuFAP: a platform for gene functional analysis in *Dendrobium huoshanense*

**DOI:** 10.1186/s12864-024-10220-6

**Published:** 2024-04-04

**Authors:** Qiaoqiao Xiao, Qi Pan, Jun Li, Jinqiang Zhang, Jiaotong Yang

**Affiliations:** Resource Institute for Chinese and Ethnic Materia Medica, Guizhou University of Traditional Chinese Medicine, 550025 Guizhou, China

**Keywords:** Platform, *Dendrobium huoshanense*, Gene function analysis, Gene Set enrichment analysis

## Abstract

**Background:**

*Dendrobium huoshanense*, a traditional medicinal and food plant, has a rich history of use. Recently, its genome was decoded, offering valuable insights into gene function. However, there is no comprehensive gene functional analysis platform for *D. huoshanense*.

**Result:**

To address this, we created a platform for gene function analysis and comparison in *D. huoshanense* (DhuFAP). Using 69 RNA-seq samples, we constructed a gene co-expression network and annotated *D. huoshanense* genes by aligning sequences with public protein databases. Our platform contained tools like Blast, gene set enrichment analysis, heatmap analysis, sequence extraction, and JBrowse. Analysis revealed co-expression of transcription factors (C2H2, GRAS, NAC) with genes encoding key enzymes in alkaloid biosynthesis. We also showcased the reliability and applicability of our platform using Chalcone synthases (CHS).

**Conclusion:**

DhuFAP (www.gzybioinformatics.cn/DhuFAP) and its suite of tools represent an accessible and invaluable resource for researchers, enabling the exploration of functional information pertaining to *D. huoshanense* genes. This platform stands poised to facilitate significant biological discoveries in this domain.

**Supplementary Information:**

The online version contains supplementary material available at 10.1186/s12864-024-10220-6.

## Background

*Dendrobium huoshanense*, a traditional medicinal and food homologous plant, is a member of the Orchidaceae family and has a rich history of medicinal use [1]. It is commonly employed for its beneficial effects on the stomach, fluid production, heat clearance, and yin nourishment [2, 3]. Previous studies have demonstrated the diverse activities of *D. huoshanense*, including immunoregulation, anti-oxidation, anti-cataract, anti-glycation, anti-aging, anti-tumor, anti-rheumatoid arthritis, anti-atherosclerosis, anti-inflammation, hypoglycemic, and liver protection activities [4–6]. The therapeutic effects are primarily attributed to active substances such as flavonoids, alkaloids, sesquiterpenes, and especially polysaccharides, which serve as the quality evaluation index for *D. huoshanense* [7, 8].

The advancement of high-throughput sequencing technology has significantly expanded research methods in the field of life sciences. This technology not only enhances the efficiency of scientific research but also drives the progress of basic research. Whole genome sequencing has been successfully accomplished in model plants and crops, with many species now possessing gene function analysis platforms that integrate multiple omics data. For instance, Tian et al. developed the MCENet platform [9], which included extensive Zea mays gene co-expression networks constructed from transcriptomic data, as well as gene function analysis tools, facilitating the study of gene function and interactions between different genes. More recently, Wang et al. analyzed genomics data from 13 species in 9 genera of the Malvaceae family and established a functional genomic hub for Malvaceae plant [10], including genome-wide association analysis (GWAS) and single nucleotide mutation site (SNP) information, along with 374 sets of transcriptomic and proteomic data.

Currently, there are few analysis platforms that include the genetic information and functionality of *D. huoshanense*. The IMP provides genome information for *D. huoshanense* [11]. However, it lacks information such as expression data, co-expression networks, and other transcriptome-related details. Many databases are not suitable for gene functional analysis of *D. huoshanense*. For instance, essential plant databases like Phytozome [12] do not include the genome and transcriptome data for *D. huoshanense*. As *D. huoshanense* possesses active ingredients with significant pharmacological effects, exploring the genes regulating these active components is crucial for researchers to obtain detailed gene information using existing platforms. Therefore, it is essential to develop a gene function analysis platform for *D. huoshanense* by integrating various annotations. Such a platform will contribute to deeper gene function analysis and exploration in this species.

In 2020, the whole genome sequencing of *D. huoshanense* was successfully completed [13]. This achievement has led to the accumulation of valuable transcriptome data for *D. huoshanense*. To fully utilize and leverage this data, we curated transcriptome data obtained from the Sequence Read Archive (SRA) at the National Center for Biotechnology Information (NCBI) and the Genome Sequence Archive (GSA) at the National Genomics Data Center (NGDC) [14]. We constructed a comprehensive co-expression network of *D. huoshanense*. Additionally, we have developed a gene function analysis platform for *D. huoshanense*, named DhuFAP. This platform incorporates various analysis tools, including BLAST, GSEA, and JBrowse, and so on. These tools are designed to facilitate the exploration of novel gene functions in *D. huoshanense* and enable researchers to delve deeper into the molecular mechanisms underlying its unique characteristics.

## Materials and methods

### Data resource

The genomic data were sourced from the CNSA database’s FTP public service, which included genome sequences, gene structure annotation files, protein sequences, and transcript sequences. Transcriptomic data were obtained from SRA and NGDC. Protein sequences from public platforms were downloaded from NCBI, Uniprot, and TAIR databases. KEGG and GO annotation information was sourced from the KEGG database and agriGO v2. The EAR protein sequences, CAZy protein sequences, and transporter protein sequences in gene families were obtained from the PlantEAR, CAZy database, and TransportDB, respectively.

### Function annotation

By utilizing the Diamond Blastp algorithm (v2.0.14.152) with the parameters “--evalue 1E-3” and “--top 1” [15], the protein sequences of *D. huoshanense* were aligned to those present in public databases, including NR, Uniprot, SwissProt, and TAIR. The resulting annotation information was obtained from the best match identified in these databases. KEGG annotation was performed using the GhostKOALA website [16], and the predicted KEGG numbers were employed to retrieve annotation information from the KEGG database [17]. GO annotation and pfam domain information was accomplished through the InterProScan software [18], enabling the acquisition of GO numbers. Subsequently, the corresponding annotation information was downloaded from agriGO v2.0 [19] based on the obtained GO numbers.

### Co-expression network construction

Downloaded transcriptome samples were mapped to the reference genome of *D. huoshanense* using the Hisat2 software [20], resulting in alignment SAM files. Subsequently, SAM files were converted to BAM file and sorted using sam tools [21]. The stringtie software [22] was then employed to obtain the expression values for each transcriptome sample, enabling the construction of an expression matrix. Using the PCC algorithm, we calculated the correlation between gene expressions for every pair of genes. The gene correlations were subsequently ranked using the MR algorithm. The formula is as follows:

$$ PCC=\frac{\sum (X-\stackrel{-}{X})(Y-\stackrel{-}{Y})}{\sqrt{{\sum }_{i=1}^{n}{({X}_{i}-\stackrel{-}{X})}^{2}}\sqrt{{\sum }_{i=1}^{n}{({Y}_{i}-\stackrel{-}{Y})}^{2}}}$$

$$ MR\left(AB\right)=\sqrt{Rank\left(AB\right)\times Rank\left(BA\right)}$$

In the given formulas, ‘n’ represents the total number of samples in the RNA-seq data, while ‘x’ and ‘y’ represent the TPM values. The term ‘Rank’ refers to the order of PCC values, where ‘AB’ signifies the ranking of gene A among all genes with gene B, and ‘BA’ indicates the reverse ranking.

Gene pairs in co-expression networks have similar expression patterns and may therefore have similar functions. Similar functionality can be evaluated by GO. The more similar the GO between co-expressed gene pairs, the more reliable the co-expression network will be. We used co-expressed genes to assess whether the GO can be accurately predicted. If an accurate prediction is true, it cannot be accurately predicted to be false. We took these predictions as input to a binary classifier and calculated the true positive rate (TPR) and false positive rate (FPR), and then ploted the ROC curve. The greater the area under the curve (AUC) values, the better the prediction effect and the more robust the co-expression network. We identified Gene Ontology (GO) terms associated with biological processes, with a particular focus on those exhibiting gene counts ranging from 4 to 20. We evaluated the areas under the ROC curve (AUC) at different thresholds. By comparing the AUC values, we determined the property PCC and MR thresholds.

### Protein-protein interaction (PPI) network

The construction of the PPI network for *D. huoshanense* involved the use of the OrthoFinder softwareb [23] to predict orthologous relationships between *Arabidopsis* and *D. huoshanense*. Subsequently, the PPI network was mapped from *Arabidopsis* to *D. huoshanense*, establishing the PPI network in *D. huoshanense*.

### Gene family identification

Initially, a hidden Markov model obtained from iUUCD 2.0 [24] successfully identified ubiquitin families in *D. huoshanense*. The log-odds likelihood scores parameter was from the threshold recommended by the iUUCD 2.0 [24]. OrthoFinder [23] was employed for the prediction of orthologous relationships between *Arabidopsis* and *D. huoshanense* with the default parameter. Following this, identification of proteins with TP, CAZy and proteins with EAR motifs were carried out utilizing the established orthologous relationship. The iTAK software [25] was utilized to identify transcription factors and protein kinases in *D. huoshanense* and the command was “iTAK.pl + protein_sequence”. The complete genome underwent KEGG pathway annotation through the utilization of GhostKOALA [16]. Moreover, an analysis of the functional annotations for CYP450 genes was carried out, utilizing the information provided by KEGG annotations.

### Construction of DhuFAP

The platform was built using the LAMP (Linux, Apache, MySQL, PHP) technical stack as its foundation. A MySQL database was created by importing various results and data analyses, such as gene structure annotation, co-expression network, gene functional annotation, PPI network, and gene family information. To enhance data visualization, responsive websites were created by employing a combination of HTML, PHP, JavaScript, and CSS programming languages.

### Toolkit for gene function analysis

We integrated Gene Set Enrichment Analysis (GSEA) [26], building upon previous descriptions [27–29]. We also incorporated JBrowse software [30], a tool developed by Buels et al., to display transcriptome data and blast tools [31] to find similar sequences. Furthermore, we introduced a sequence extraction tool using a Perl script and implemented a Heatmap analysis tool based on Highchart Javascript. These additions expanded the capabilities of the platform and improved the visualization and analysis of data.

## Result

### Gene functional annotation

**Fig. 1:**
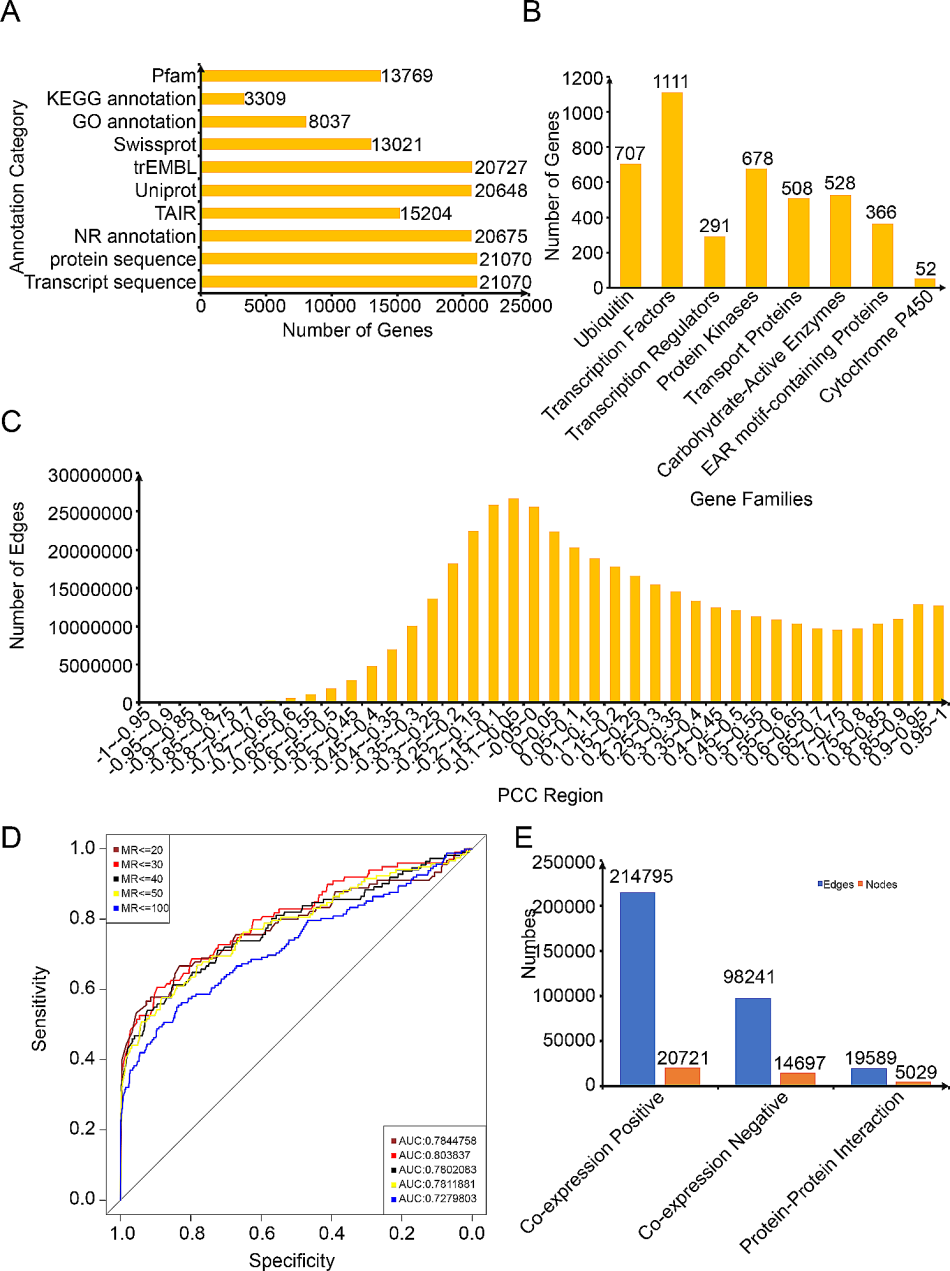
Related statistical information of DhuFAP. (**A**) Gene function annotation information provided by DhuFAP. (**B**) Gene family classification information available. (**C**) The relationship between Pearson correlation coefficient (PCC) and the number of edges in the co-expression network. (**D**) Distribution of Area Under the Curve (AUC) values at different Matural Rank (MR) thresholds. (**E**) Statistical analysis of nodes and edges in the positive co-expression network, negative co-expression network, and Protein-Protein Interaction (PPI) network

We obtained the genome data of *D. huoshanense* from NGDC, which included a comprehensive dataset of 21,070 transcripts and 21,070 proteins. To ensure accurate annotation, we subjected these resources to alignment with the protein sequences against well-known databases such as NR, Uniprot, TAIR, trEMBL, and Swissprot. Consequently, we annotated a total of 20,675, 20,648, 15204, 20,727, and 13,021 genes, respectively. Furthermore, we utilized InterProScan software to conduct Gene Ontology (GO) annotations on a total of 8,037 genes [18]. For a comprehensive understanding of functional pathways, we utilized the GhostKOLAL [16] online tools to map KEGG annotation onto a set of 3,309 genes. Lastly, we conducted functional characterization of protein domains using the PfamScan software [32] to provide a comprehensive understanding of proteins (Fig. 1A).

### Gene family classification

Initially, using iTAK software, we identified 1,111 transcription factors (TFs), 291 transcription regulators (TRs), and 678 protein kinases (PKs) in *D. huoshanense* respectively. Subsequently, utilizing the HMM profile derived from the ubiquitin-proteasome dataset within the iUUCD v2.0 database, we predicted 707 genes accountable for encoding components within the ubiquitin-proteasome system. Additionally, through gene alignment with databases like PlantEAR, TransprotDB, and CAZy, we effectively pinpointed 366 EAR genes, 508 genes to the Transprot family, and 528 genes categorized under the CAZy family. In addition, KEGG annotation allowed us to anticipate the existence of 52 Cytochrome P450 genes (Fig. 1B). These analyses provided valuable insights into the transcriptional regulation, protein kinase activity, ubiquitin-proteasome system, and gene families present in *D. huoshanense*.

### Construction of co-expression network

We collected 69 transcriptome samples from SRA and NGDC, encompassing data from diverse tissues (roots, stems, leaves) under normal growth conditions and various treatments (drought, low temperature, MeJA) under environment stress. Then we constructed a co-expression network by utilizing these transcriptome data, which were subsequently mapped to the reference genome with a mapping ratio exceeding 60% (Table S1). We analyzed the Pearson correlation coefficient (PCC) values obtained from expression profiles to identify co-expressed gene pairs. Many gene pairs showed no or weak correlation in their expression patterns (Fig. 1C). To pinpoint gene pairs closely linked within each other’s network, we used the MR (Matural Rank) method based on their PCC ranking values.

To ensure the reliability of our constructed network, we selected GO terms associated with similar biological activities, resulting in 120 terms with varying gene counts ranging from 4 to 20. We compared the area under the curve (AUC) values for different PCC (0.6, 0.7, 0.8, 0.9), considering the overlap between positively co-expressed genes and the previously selected GO gene sets. We observed non-significant differences in AUC values among the PCC networks. To encompass a broader set of genes, we opted for a PCC threshold of > 0.6 (Figure S1). We further examined the area under the curve (AUC) values across various MR thresholds with the constraint of PCC > 0.6. This analysis led us to establish a network threshold of MR < 30 for the positive co-expression network. The thresholds for the negative co-expression network were set at PCC<-0.5 and MR < 30 (Fig. 1D). The resulting co-expression network for *D. huoshanense* consisted of 313,036 co-expression gene pairs. This contained 214,795 gene pairs in the positive co-expression network and 98,241 gene pairs in the negative co-expression network (Fig. 1E).

### Protein–protein interaction network

By predicting the orthologous genes between *Arabidopsis* and *D. huoshanense*, we mapped the protein-protein interaction (PPI) network of *Arabidopsis* onto *D. huoshanense*. This resulted in the identification of 19,589 pairs of PPI relationships, involving a total of 5,029 genes (Fig. 1E).

### DEGs in different transcriptome

In order to incorporate gene co-expression and protein-protein interaction (PPI) networks with gene expression data, we performed differential expression analysis on the transcriptome data (student’s t-test (*p* < 0.05) and fold change [|log_2_(foldchange)| > 1]), resulting in the identification of differentially expressed genes (DEGs) across five sets of data. Through this process, we obtained a total of 35 distinct groups of DEGs (Table S2).

### Platform content

**Fig. 2:**
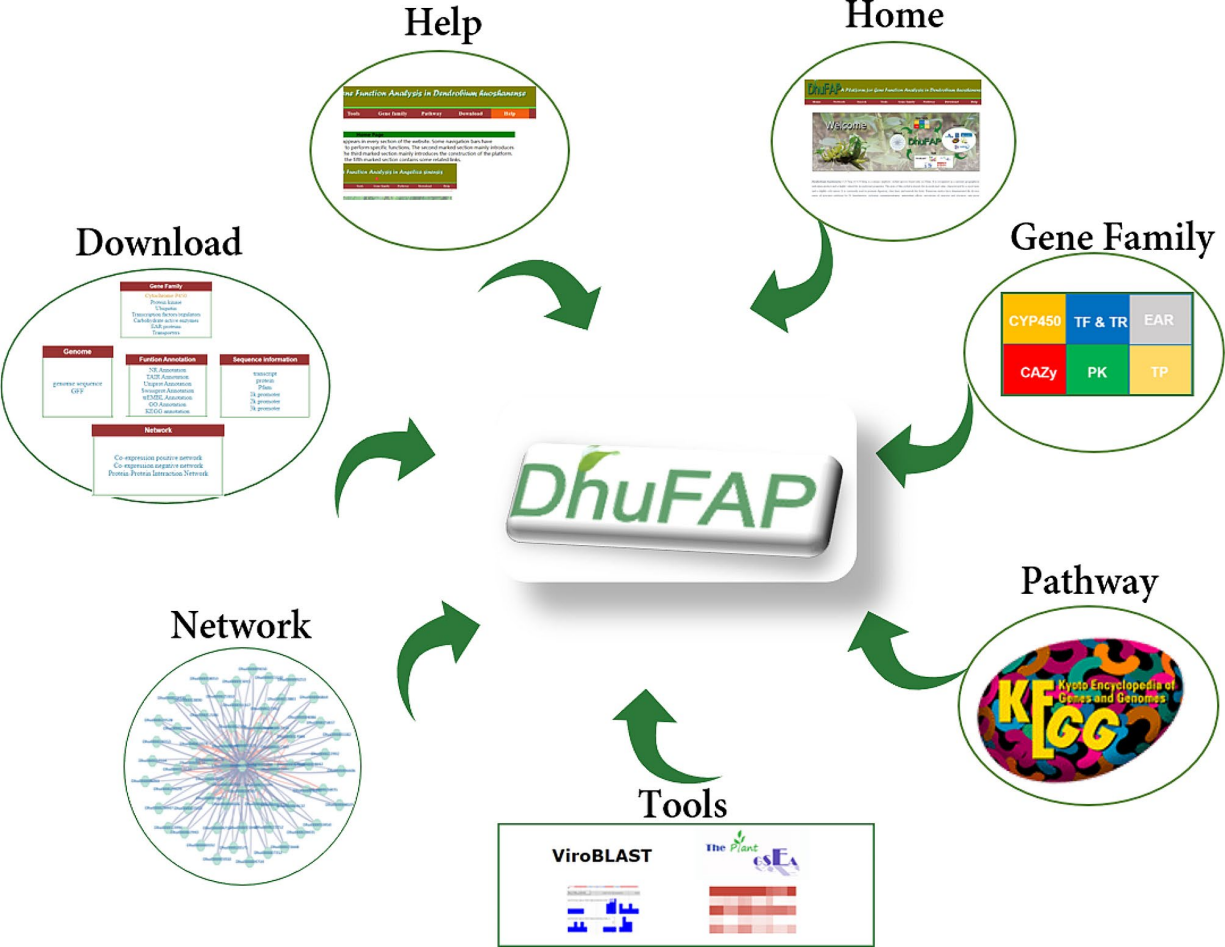
The structure of DhuFAP framework consisted of seven primary sections. The Home section served as an introduction to this platform. The Network section encompassed the co-expression network and PPI network. The Gene Family section comprised various gene families such as CYP450 family genes, transcription factors, transcription regulators, protein kinases, ubiquitin proteasomes, CAZy genes, Transport Proteins, and EAR motif-containing proteins. The Tools section offered functionalities like Search, BLAST, JBrowse, Sequence extraction, and GSEA toolkit. Pathway, Download, and Help were presented as separate sections

To enhance gene functional analysis in *D. huoshanense*, a comprehensive platform called DhuFAP has been developed. DhuFAP comprises seven sections—Home, Network, Pathway, Tools, Gene Family, Download, and Help—aimed at enhancing user-friendliness and delivering valuable insights to researchers (Fig. 2). Within the Network section, users can access both protein-protein interaction (PPI) and co-expression networks, offering comprehensive insight into the intricate molecular interactions within *D. huoshanense*. Pathway section consists of gene annotations from the KEGG database. The Gene Family section contains diverse protein families like CYP450, TF, TR, PK, TP, Ubiquitin, GAZy, and EAR motif-containing proteins. The Tools section offers various helpful features. The Blast tool screens nucleic acid or protein sequences for similarities within our database. GSEA enables comprehensive gene set enrichment analysis. The Extract Sequence tool retrieves gene sequences using accession numbers and locations. Moreover, the Heatmap Analysis tool visually displays gene expression data. The inclusion of JBrowse provides an intuitive visualization of genomic and transcriptomic features. Download section provides convenient access to relevant information, ensuring easy retrieval of necessary resources. Furthermore, the Help section offers a comprehensive user manual, guiding researchers through the platform’s functionalities and optimizing their usage of DhuFAP.

### Function application

#### Analysis of key enzyme genes in alkaloid biosynthesis pathway

The stem contains an alkaloid that is the primary bioactive component in *D. huoshanense*. According to KEGG annotation in DhuFAP, there were 34 genes associated to alkaloid biosynthesis pathways were screened (Table S3). In order to better understand the relationship between key enzyme genes in alkaloid biosynthesis and TFs, co-expression analysis was conducted to identify the TFs which expressions were correlated with the key enzyme genes. The result demonstrated that C2H2, GRAS, NAC and other TFs were co-expressed with these key enzyme genes (Figure S2 and Table S4). Co-expressed genes have the same expression pattern, and may be regulated by the same upstream transcription factors. To explore transcription factors that may bind to the key enzyme gene promoter regions, we analyzed the co-expression relationships among key enzymes. The result showed that there were four pairs of co-expression between the key enzymes (Figure S3). We extracted 3000 bp sequences from the promoter region of each co-expression module to predict transcription factor binding sites. Many transcription factor binding sites were found, including MYB, GRAS and C2H2. This suggests that these transcription factors may bind to the key enzyme promoter regions. Therefore, these transcription factors may play a crucial regulatory role in the biosynthesis of alkaloids.

#### Characteristic and functional analysis of CHS gene

**Fig. 3:**
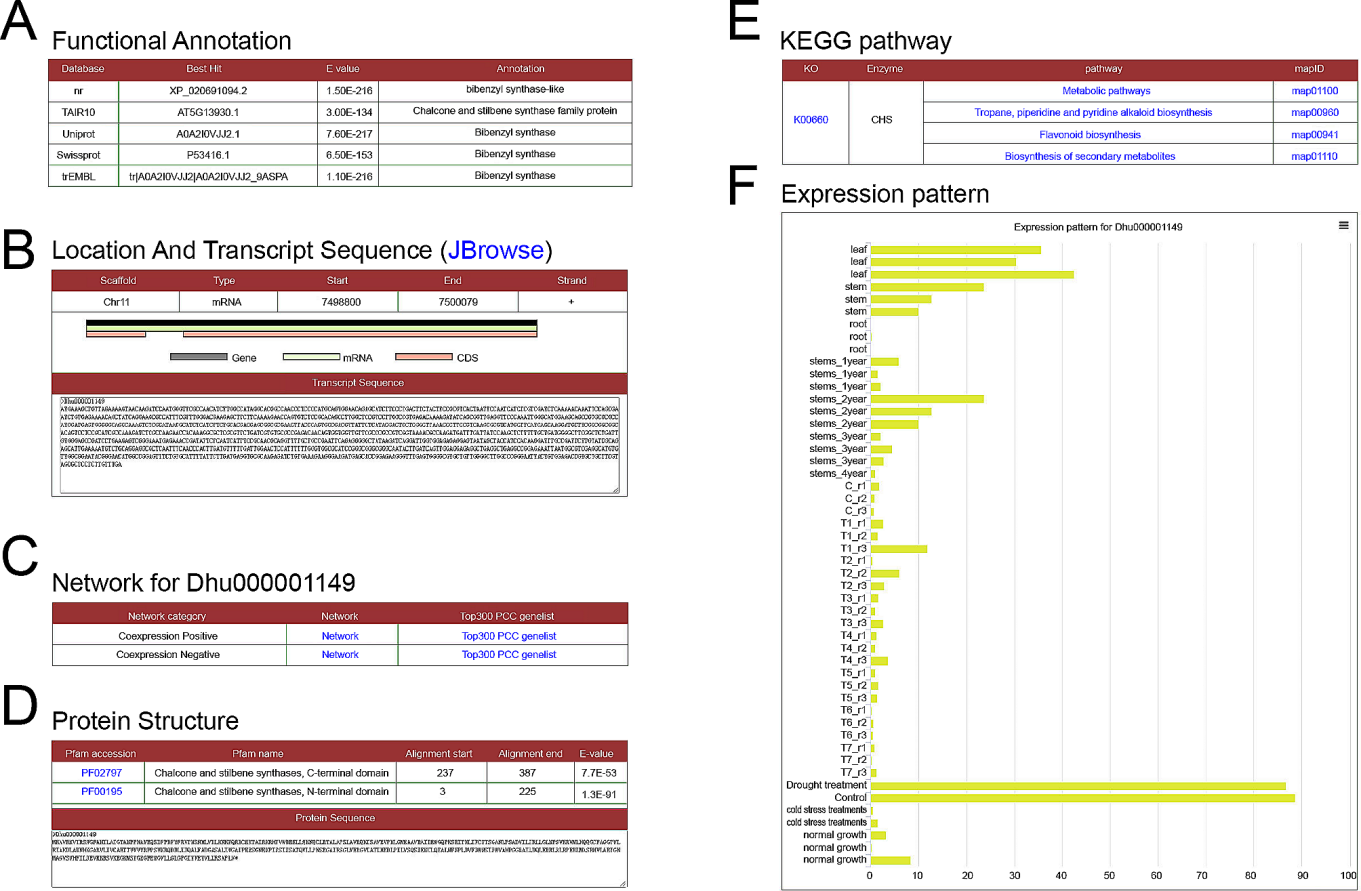
Gene details of CHS. (**A**) Functional annotations. (**B**) Location and transcript sequences. (**C**) Links for network. (**D**) Protein structure. (**E**) KEGG pathway and (**F**) Expression pattern of CHS gene

**Fig. 4:**
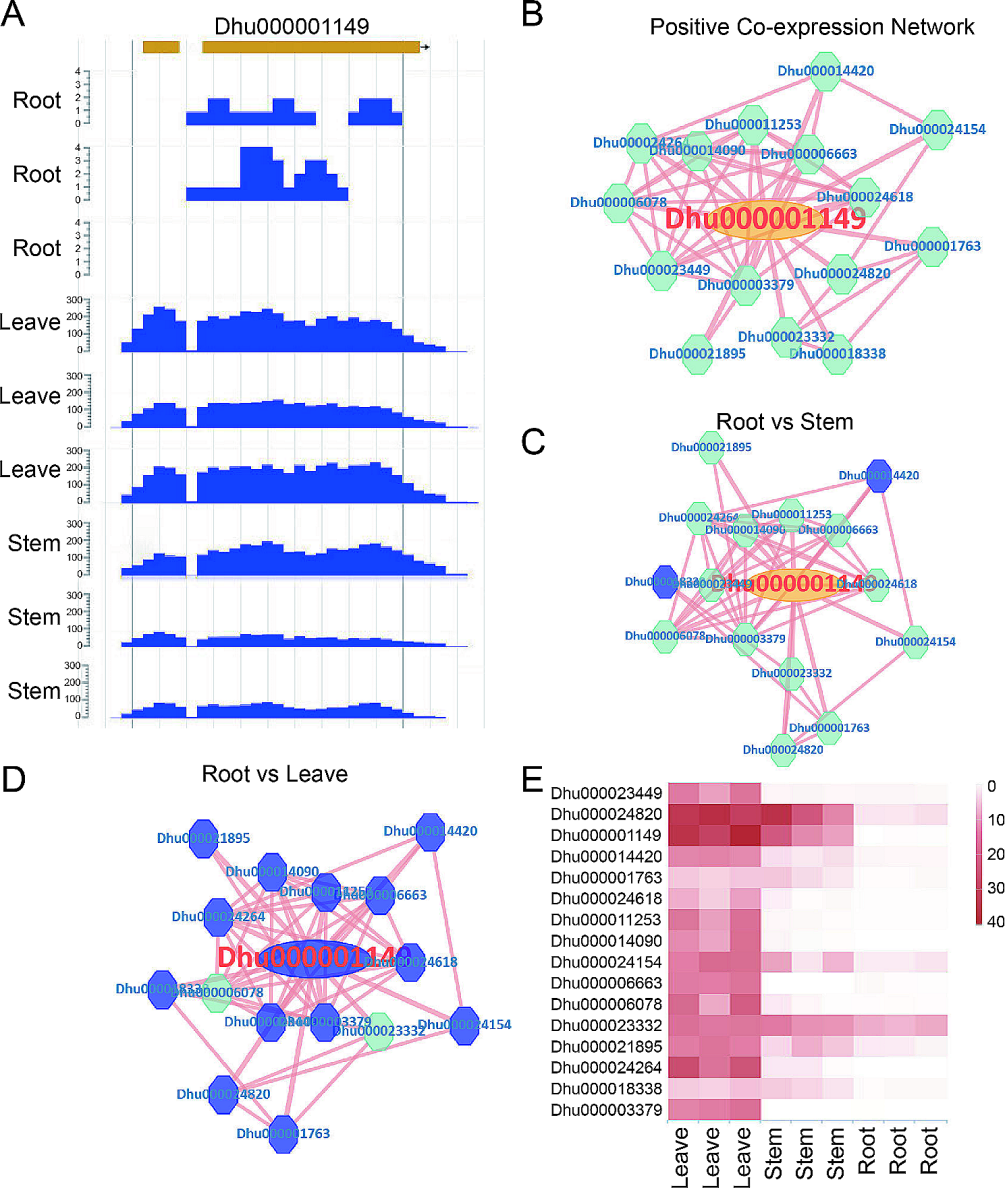
Expression and co-expression network analysis of CHS. (**A**) Presentation of CHS gene expression using JBrowse. (**B**) Positive co-expression gene network of CHS. (**C**) Analysis of gene differential expression in the positive co-expression network when comparing root and stem transcriptomes. (**D**) Analysis of gene differential expression in the positive co-expression network when comparing root and leaves transcriptomes. (**E**) Comparative analysis of CHS co-expressed genes’ expression in different transcriptome samples using heatmap analysis tool

Chalcone synthases (CHS) are key enzyme that catalyzes alkaloid biosynthesis [33]. In our platform, the gene *Dhu000001149* was identified as a member of chalcone synthase family (Fig. 3A), spans from 7,498,800 to 7,500,079 bp on chromosome 11 (Fig. 3B). Additionally, co-expression network connections were also furnished (Fig. 3C). It was found that Chalcone and stilbene synthases domain was located at N-terminal and C-terminal in the protein sequence (Fig. 3C). KEGG annotation suggested that enzyme (Fig. 3D and E) involved in tropane, piperidine and pyridine alkaloid biosynthesis and flavonoid biosynthesis. Previous studies have identified CHS involved in the potential accumulation of alkaloid in *D. huoshanense* [34]. Through expression profiling analysis, we found that the expression level of this gene was higher in stem and leave compared to root (Fig. 3F). The display of reads mapping using JBrowse also revealed higher expression in leaf and stem (Fig. 4A). Furthermore, the accumulation of alkaloid significantly higher in stem, leaf than root [7]. The expression of this gene showed a similar trend to the synthesis and accumulation of active compounds. Therefore, the analysis results suggest that the gene may be involved in the accumulation of alkaloid.

Furthermore, we conducted a co-expression analysis of CHS with its expression profiles. Network analysis revealed 15 genes that showed positive co-expression with CHS (Fig. 4B, Table S4). Additionally, many genes in the co-expression network were significantly upregulated in the leaves and stem (Fig. 4C and D). Analysis of co-expressed genes with CHS through heatmap analysis also revealed similar results (Fig. 4E). Therefore, our analysis suggests that the CHS gene plays an important role in regulating biosynthesis of alkaloid.

#### Comparative transcriptome analysis

**Fig. 5:**
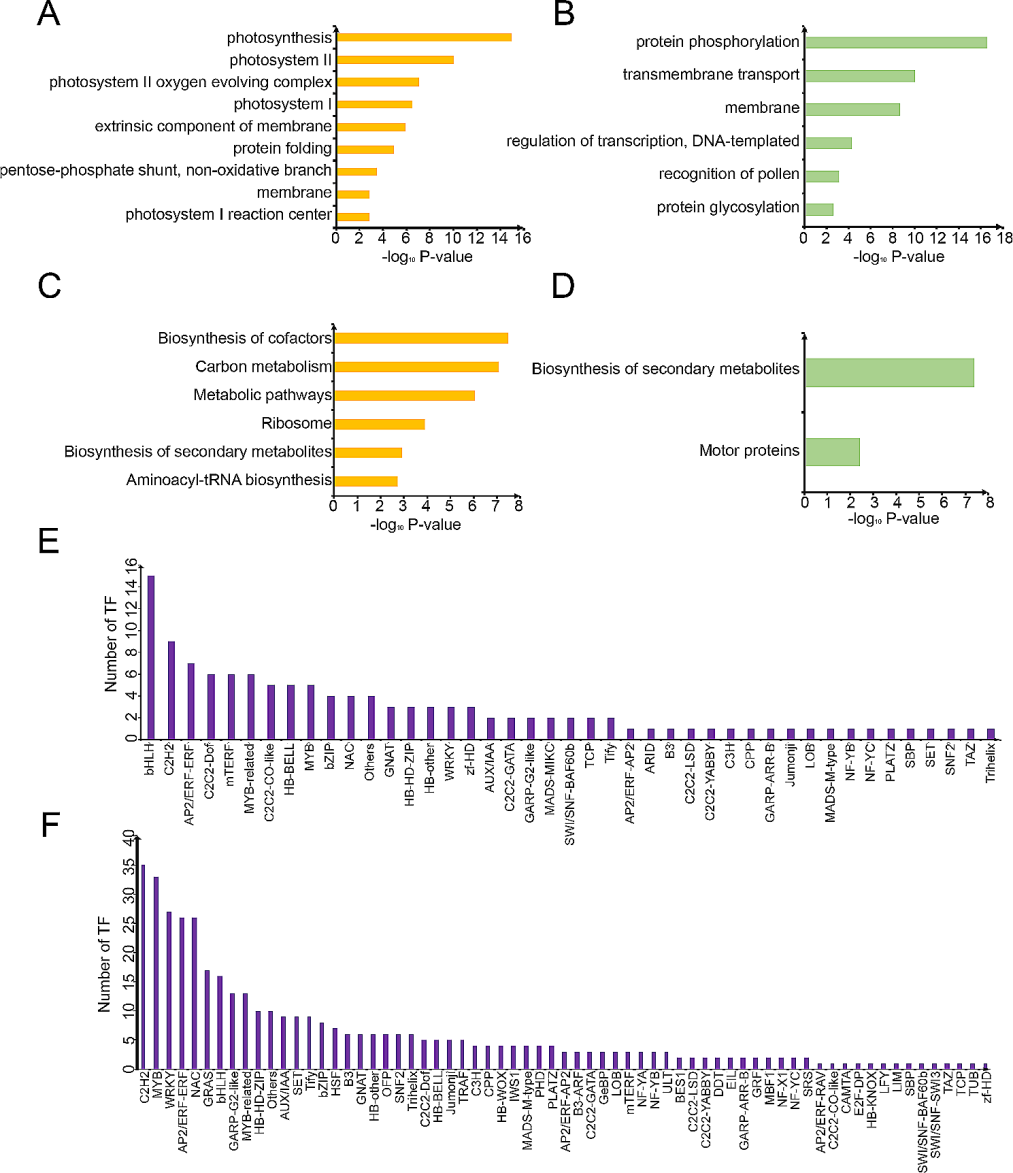
Analysis of root and leaf samples from a set of transcriptome data (CRA000551). (**A**) Results of GO enrichment analysis for significantly upregulated genes. (**B**) Results of GO enrichment analysis for significantly downregulated genes. (**C**) Results of KEGG enrichment analysis for significantly upregulated genes. (**D**) Results of KEGG enrichment analysis for significantly downregulated genes. (**E**) Presence of transcription factors in significantly upregulated genes. (**F**) Presence of transcription factors in significantly downregulated genes

In order to uncover potential key regulatory factors involved in alkaloid biosynthesis, this study conducted an analysis on a transcriptome dataset (CRA000551), including leaves and root with 3 replicates. Using student’s t-test (*p* < 0.05) and fold change [|log_2_(foldchange)| > 1], we identified 1633 up-regulated and 4387 down-regulated genes by comparing the transcriptomes of roots and leaves (Table S2). Using the GSEA analysis tool provided by the platform, we performed GO enrichment analyses for the up-regulated and down-regulated genes. For the up-regulated genes, there was a significant enrichment of genes related to protein folding and photosystem (Fig. 5A). For the down-regulated genes, there was a significant enrichment of genes related to protein phosphorylation, transmembrane transport, and regulation of transcription (Fig. 5B). We also performed KEGG enrichment analysis and found that pathways related to biosynthesis of cofactors and carbon metabolism, and biosynthesis of secondary metabolites were significantly enriched for the up-regulated genes in root (Fig. 5C). On the other hand, pathways related to biosynthesis of secondary metabolites were significantly enriched for the down-regulated genes in root (Fig. 5D). Therefore, these enrichment analysis results suggest that these differentially expressed genes may play a role in the synthesis of secondary metabolites, including alkaloids. Additionally, we focused on analyzing the transcription factors among these genes. Among the up-regulated gene set, there were a higher number of transcription factors such as AP2/ERF-ERF, C2H2, and bHLH (Fig. 5E). In contrast, the down-regulated gene set had a higher occurrence of transcription factors such as NAC, WRKY, MYB, and C2H2 (Fig. 5F). Since alkaloid synthesis is significantly higher in leaves compared to roots [7], the identified transcription factors may play a role in regulating the process of alkaloid biosynthesis.

## Discussion

We have developed a comprehensive gene function analysis platform, DhuFAP, specifically designed for *D. huoshanense*. Our platform aims to provide researchers with a wide range of resources and tools to gain deeper insights into the functional genes and related biological processes of this species. Compared to other platforms, our platform is more professional and comprehensive, and can meet the diverse needs of researchers. DhuFAP focuses on *D. huoshanense* with specific genome annotation and functional analysis. This allows researchers to gain a deeper understanding of the gene function of this traditional medicinal and food plant. Compared to generic platforms, we better meet the research needs of specific plants.

To further elucidate gene functions within the co-expression network, our platform offers various analysis tools. These tools include gene set enrichment analysis, regulatory network analysis, gene expression pattern analysis, and more. Users can leverage these tools according to their research needs to uncover the biological significance within the co-expression network. To facilitate effective platform utilization, we provide detailed usage examples that demonstrate how to analyze functional genes in DhuFAP. These examples showcase key steps such as filtering out important genes from the co-expression network, performing gene enrichment analysis, and interpreting regulatory networks. They not only highlight the capabilities of the platform but also offer practical guidance for users to conduct their own analyses.

DhuFAP serves as a powerful tool for researchers to delve into the functional genes and related biological processes of *D. huoshanense*. By integrating co-expression networks and offering various analysis tools, along with detailed usage examples, we are committed to advancing *D. huoshanense* research and providing valuable resources for scientists in related fields.

While DhuFAP offers valuable features and tools, we acknowledge potential limitations and areas for improvement. We predicted the protein interaction network of *D. huoshanensis*. The more protein interaction pairs that have been experimentally confirmed, the more accurate our PPI predictions will be. Compared the predicted interaction protein with those have been reported, the more overlapping the predicted interaction protein is, the more reliable predicted protein interaction network is. We investigated the literature of *D. huoshanense* and found that no protein interaction research of *D. huoshanense* had been reported. If relevant research reported in the future studies, we will further evaluate the predicted protein interaction network.

Currently, the platform relies on existing gene expression datasets, and ensuring data quality and coverage remains a challenge. In the future, we plan to expand the scale and diversity of the dataset to provide more comprehensive and accurate analysis results. Additionally, we aim to refine and expand the analysis tools and functionalities of the platform. This involves continuous improvement, introducing new analysis methods and algorithms, and staying updated on the latest research advancements in the field of *D. huoshanense*.

### Electronic supplementary material

Below is the link to the electronic supplementary material.

Supplementary Material 1

Supplementary Material 2

## Data Availability

All the data we used are sourced from public platforms. The genome sequences analysed during the current study are public available in China National GeneBank (CNGB)(https://ftp.cngb.org/pub/CNSA/data3/CNP0000830/CNS0251991/CNA0014590/). Transcriptome data are publicly available in Sequence Read Archive (SRA) database (Accession no: SRP122499, SRP151171, SRP225982, SRP268245, SRP291861 and SRP406621) and GSA database in National Genomics Data Center (NGDC) (Accession no: CRA000551, CRA005817 and CRA006607).
