# Supplementary material for: DhuFAP: a platform for gene functional analysis in Dendrobium huoshanense
Source: BMC Genomics. 2024 Apr 4;25:342. doi: 10.1186/s12864-024-10220-6 (PMC10996181; doi:10.1186/s12864-024-10220-6)
Supplement: Supplementary file 1 — Supplementary Material 1 [file 12864_2024_10220_MOESM1_ESM.docx]

***Supplementary Material***

DhuFAP: A platform for gene functional analysis in *Dendrobium huoshanense*

Qiaoqiao Xiao^1^, Qi Pan^1^, Jinqiang Zhang^1^*, Jiaotong Yang^1^*

^1^Resource Institute for Chinese and Ethnic Materia Medica, Guizhou University of Traditional Chinese Medicine, Guizhou 550025, China.

**Supplementary Figures**

**
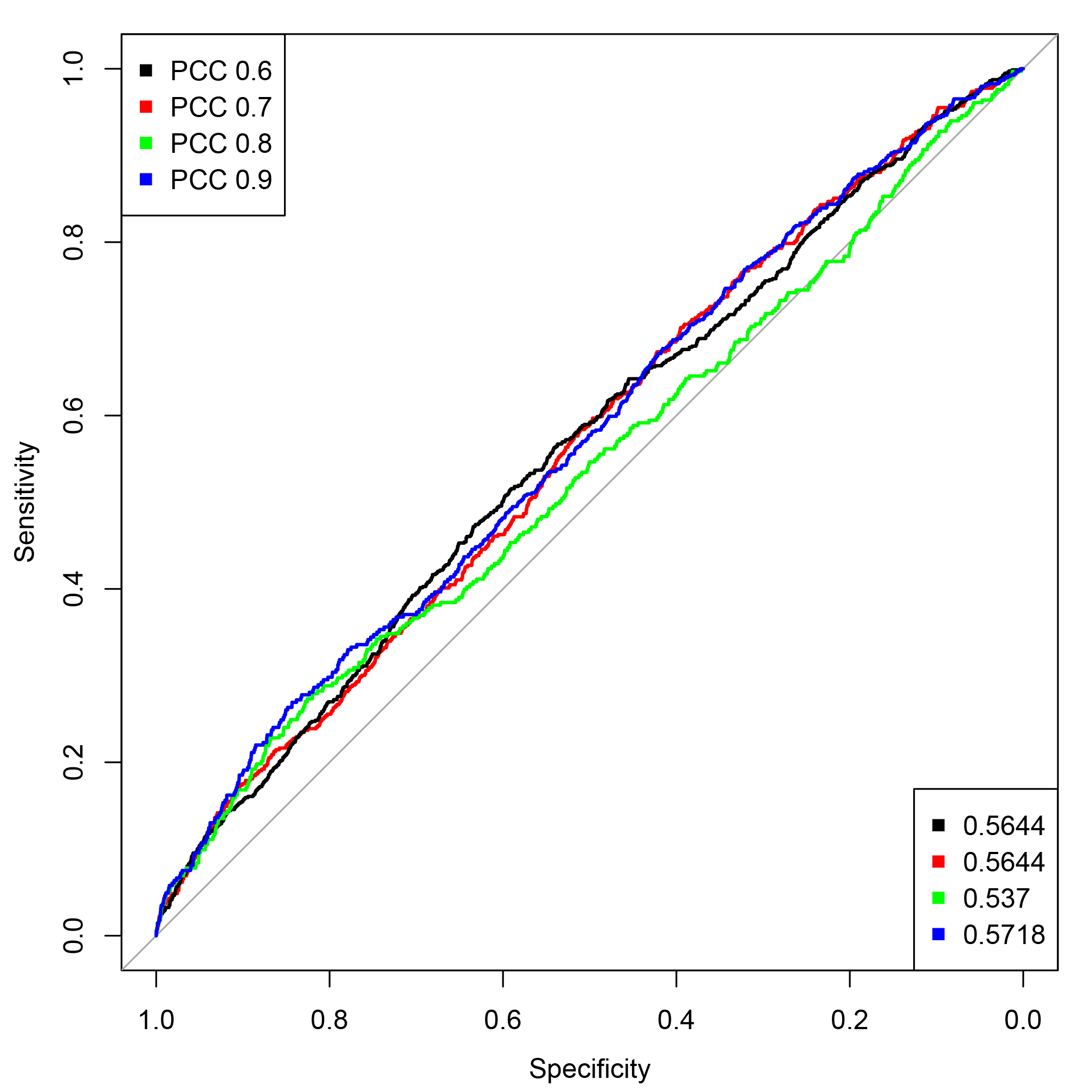
**

**Figure S1** The AUC value of the co-expression network under different PCC values.


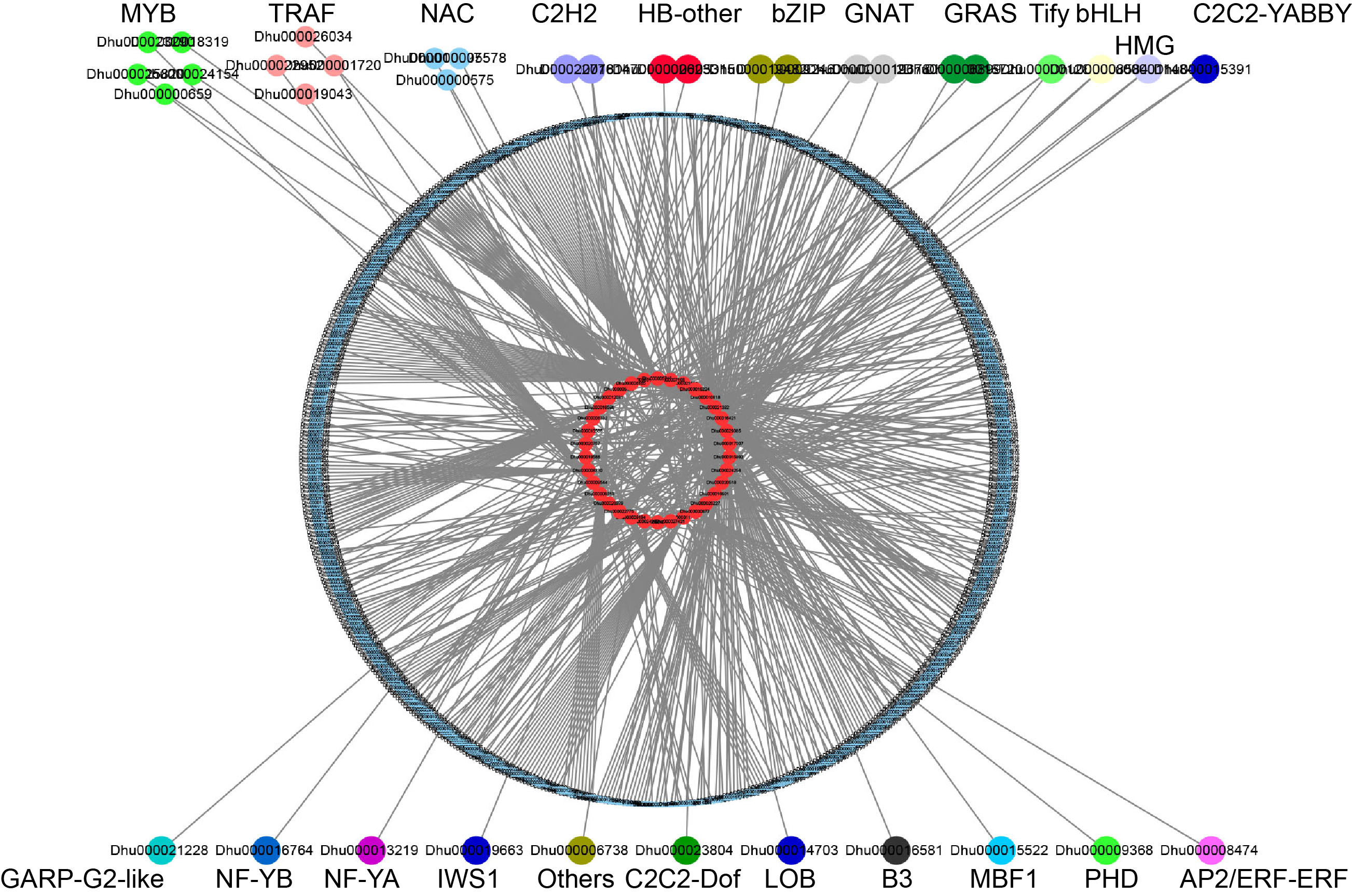


**Figure S2** Co-expression relationship between TFs and key enzyme genes in alkaloid biosynthesis.





**Figure S3** Co-expression relationship between key enzyme genes in alkaloid biosynthesis and promoter TF binding sites analysis. (A) Number of shared transcription factor binding sites in the first pair of co-expression relationships and promoter regions.(B) Number of shared transcription factor binding sites in the second pair of co-expression relationships and promoter regions.(C) Number of shared transcription factor binding sites in the third pair of co-expression relationships and promoter regions.(D) Number of shared transcription factor binding sites in the fourth pair of co-expression relationships and promoter regions.
